# Influence of doctor-patient trust on the adoption of mobile medical applications during the epidemic: a UTAUT-based analysis

**DOI:** 10.3389/fpubh.2024.1414125

**Published:** 2024-08-19

**Authors:** Dong Meng, Zhenyi Guo

**Affiliations:** School of Media and Communication, Shanghai Jiao Tong University, Shanghai, China

**Keywords:** internet health care, COVID-19, health information management, digital health, UTAUT

## Abstract

This study examines the factors influencing users’ intention to continue using mobile medical apps within the framework of the Unified Theory of Acceptance and Use of Technology (UTAUT) model. Through a combination of questionnaire surveys and interviews, the research finds that doctor-patient trust, Performance Expectancy (PE), social influence, and facilitating conditions significantly impact users’ intention to utilize mobile medical apps. Furthermore, the study reveals the moderating effect of doctor-patient trust on social influence, indicating an increased trust level during the epidemic, attributed to positive media coverage, complimentary medical services, and risk-sharing initiatives. These results provide valuable insights for the field of internet healthcare, COVID-19 response strategies, health information management, and the advancement of digital health technologies, spotlighting the pivotal roles of trust, PE, and social influence in fostering sustained engagement with mobile health apps.

## 1 Background

Health information is a cornerstone of individual well-being, encompassing elements crucial for disease prevention, nutrition, and overall health maintenance. The advancement of communication technology has revolutionized the dissemination of health information, shaping the way individuals access and engage with crucial healthcare resources. Efforts in China to integrate health literacy initiatives reflect a broader commitment to enhancing public awareness and promoting active health information seeking among the population. The Healthy China Action has prioritized programs aimed at improving health literacy levels through tailored education and promotion strategies. Though progress has been made, continued dedication is vital to achieving the envisioned health literacy goals by 2030. The onset of the COVID-19 pandemic has accelerated the development and adoption of internet-based healthcare services, offering innovative solutions for remote care provision and the widespread sharing of health information. However, challenges persist in ensuring the quality and reliability of health communication, which significantly shapes public trust in healthcare services and information. Notably, the recent approval of internet hospitals in Shanghai underscores the escalating demand for online medical services, with notable increases in usage and service enhancements observed during the pandemic.

The dynamic landscape of communication in the new media era has introduced evolving user roles, such as group dynamics, evolving socialization patterns, and the influence of authority on user preferences and content consumption behavior. Data mining plays a crucial role in decoding user behavior patterns within content consumption and social interactions, providing invaluable insights for content creators and healthcare providers alike. The doctor-patient relationship in the digital age holds significant importance, directly impacting health communication practices and attitudes towards healthcare among the public. The portrayal of healthcare providers in Chinese media and responses to the pandemic have wielded profound influence on public perceptions of the doctor-patient relationship, highlighting the pivotal role of fostering harmonious interactions in fostering trust and support for healthcare providers through positive media coverage.

## 2 Research theory

Venkatesh et al. (1) summarized previous studies related to Technology Acceptance Model (TAM) and identified the issue of “factors influencing user cognition.” As a response to this issue, they proposed the Unified Theory of Acceptance and Use of Technology (UTAUT), also known as the authoritative model.

UTAUT consists of four core dimensions:Performance Expectancy (PE) reflects the extent to which individuals believe that using a system will enhance their job performance; Effort Expectancy (EE) measures the perceived ease of use of a system; Social Influence (SI) captures the influence of social surroundings on an individual’s technology acceptance, including subjective norms, social factors, and public image; Facilitating Conditions (FC) assesses the perception of organizational support and available resources for system use.

Moreover, UTAUT identified four significant control variables that impact the core dimensions: gender, age, experience, and voluntariness. Venkatesh et al. (1) highlighted that the combined effect of multiple control variables enhances their influence. Additionally, other theories such as Task-Technology Fit (TTF), Innovation Diffusion Theory (IDT), Theory of Reasoned Action (TRA), Theory of Planned Behavior (TPB), Motivational Model (MM), Combined TAM and TPB (C-TAM-TPB), Model of PC Utilization (MPCU), and Social Cognitive Theory (SCT) offer further explanatory power across various domains.

In addition to UTAUT, trust plays a crucial role in technology acceptance and adoption. Building trust in technology, service providers, and information security is essential for users to feel confident and willing to engage with new technologies. Trust-related theories such as Trust Theory, Social Exchange Theory, and Trust Transfer Theory can provide insights into the dynamics of trust in the context of technology adoption. By incorporating trust theories into the research framework, a more comprehensive understanding of the factors influencing mobile medical app adoption can be achieved.

## 3 Literature review

The Unified Theory of Acceptance and Use of Technology (UTAUT) model has been extensively applied in the analysis of mobile healthcare applications to understand user behavior and adoption trends. When examining mobile health apps through the lens of the UTAUT model, researchers investigate how factors such as Performance Expectancy (PE), Effort Expectancy (EE), Social Influence (SI), and Facilitating Conditions (FC) influence users’ intentions to use and continue using these applications (1).

The adoption and sustained use of mobile medical applications have become essential facets of modern healthcare, particularly highlighted during health crises such as the COVID-19 pandemic. Several studies provide insights into the factors influencing user engagement with these technologies, often grounding their findings in established behavioral frameworks. This literature review synthesizes relevant research that informs the understanding of user behaviors regarding mobile health applications, focusing on key dimensions such as trust, performance expectancy, social influence, and facilitating conditions.

Celsi and Olson (2) emphasize the role of consumer involvement in attention and comprehension processes, noting that a heightened sense of involvement leads to improved engagement and understanding of health-related information. This idea resonates with the findings of Rai et al. (3), who explore the determinants of consumer mobile health usage intentions. Their research points out that high levels of user involvement and trust in healthcare applications significantly enhance the likelihood of adoption and continued use.

Trust remains a crucial element in healthcare interactions, as highlighted by Harrison and McKnight (4), who provide a comprehensive typology of trust in e-commerce contexts. Their interdisciplinary approach underlines the multifaceted nature of trust and its impact on customer relationships, which is applicable in healthcare settings where patient trust can influence engagement with mobile medical applications. The study by Mayer et al. (5) further elucidates this point by presenting an integrative model of organizational trust, showcasing how mutual trust can foster stronger doctor-patient relationships—a critical factor emphasized in the current study’s UTAUT model application.

Cho (6, 7) investigates the adoption of smartphone health apps among college students and underscores the importance of post-adoption beliefs on the continued use of these applications. Their findings align with the current study, which identifies performance expectancy as a significant determinant of user intention. Similarly, Dutta Bergman (8) finds a correlation between health orientation and satisfaction, which underscores the necessity for healthcare providers to foster effective communication to enhance user experience and satisfaction with mobile health resources.

The impact of social influence is another vital area discussed in the literature. Zhang et al. (9) utilize a modified theory of reasoned action model to understand gender differences in m-health adoption and underscore the effects of social norms on technology acceptance. The current study extends this discussion by revealing the moderating role of doctor-patient trust on social influence, particularly in the context of heightened trust during an epidemic, driven by positive media coverage and risk-sharing initiatives.

Facilitating conditions are also pivotal to technology acceptance. The DeLone and McLean Model (10) of information systems success highlights the importance of various external factors that can affect user satisfaction and system use. The work of Shin et al. (11) on IT service success conditions complements this perspective by examining the duality of success factors in technology adoption. These insights are particularly relevant for app developers and healthcare providers aiming to improve the usability and accessibility of mobile medical applications.

In summary, the investigated literature provides a robust framework for understanding user intentions towards mobile medical applications, particularly during health crises. The studies reviewed affirm the importance of trust, performance expectancy, social influence, and facilitating conditions. As the landscape of healthcare continues to evolve with digital technologies, these elements will remain critical for fostering user engagement and improving health outcomes through mobile applications. The current study’s findings contribute valuable insights to this ongoing dialogue, reinforcing the need for a focus on trust and user experience in digital health strategies.

## 4 Research methods

### 4.1 Survey methodology

This study adopts a mixed-methods approach to achieve the research objectives. Quantitative methods were employed to design, distribute, collect, and analyze questionnaires that aimed to investigate the public’s utilization of Internet-based medical services during the current epidemic.

Participants were asked to respond to the following statements on a Likert scale ranging from 1 (Strongly Disagree) to 5 (Strongly Agree):

I have experienced a prolonged period of epidemic lockdown.
My family members have experienced a prolonged period of epidemic lockdown.
My region has experienced a prolonged period of epidemic lockdown.
Doctors prioritize my needs.
I follow the advice given by doctors.
I believe what doctors tell me is true.
I trust doctors’ judgments about my condition.
I believe doctors consider all factors in diagnosis and treatment.
If there are errors in diagnosis or treatment, doctors will inform me.
Using mobile medical apps helps improve my health.
Mobile medical apps can address my health and medical needs.
Using mobile medical apps saves time in accessing medical services.
I find mobile medical apps very user-friendly.
Learning to use mobile medical apps does not take me much time.
Using mobile medical apps makes it easy for me to access health and medical resources.
My family believes I should use mobile medical apps.
My friends believe I should use mobile medical apps.
My colleagues (classmates) believe I should use mobile medical apps.
My local network is stable and allows me to use mobile medical apps freely.
My mobile network is stable and allows me to use mobile medical apps freely.
Mobile medical apps are compatible with my phone, allowing me to use them anytime, anywhere.
I am willing to use mobile medical apps again.
I am willing to recommend mobile medical apps to others. I would give positive feedback on mobile medical apps.

### 4.2 Interview methodology

This study aims to explore the influence of doctor-patient trust on the adoption of mobile medical applications during the pandemic, utilizing a qualitative research approach and conducting semi-structured interviews to gather insights and experiences from both doctors and patients. The study includes 30 participants recruited through medical institutions and online platforms. The interviews focus on themes such as doctor-patient trust, experiences with mobile medical app usage, and the impact of trust enhancement on app adoption, with the objective of gaining in-depth understanding of participants’ perspectives and experiences.

The interview outline is as follows:

1. Have you used mobile medical applications during the pandemic? What motivated you to start using these applications?
2. How do you perceive the importance of trust between doctors and patients in relation to your use of mobile medical applications?
3. Have you observed a correlation between the level of doctor-patient trust during the pandemic and your intention to use mobile medical applications? Could you share specific experiences?
4. How do you evaluate your actual user experience with mobile medical applications, and have there been any changes due to variations in trust levels?
5. Based on your personal experiences, has an increase in trust inclined you towards using mobile medical applications during the pandemic? Why?
6. How do you think the doctor-patient trust established during the pandemic will affect your future intention to use mobile medical applications?
7. In your use of mobile medical applications, how do you believe doctor-patient trust influences your user experience and sense of trust?
8. Have you noticed any impact of changes in medical services during the pandemic on doctor-patient relationships and the use of mobile medical applications?
9. How do you think trust between doctors and patients influences your intention to continue using mobile medical applications?
10. Have your perceptions of trust towards doctors and healthcare institutions currently providing medical services changed? How has this affected your views on mobile medical applications?

Data collection was completed in the first half of 2023, with interview content recorded in full through audio recordings and text transcripts. During the data analysis phase, qualitative analysis methods were employed to organize and summarize the interview content, aiming to uncover potential associations and influencing factors between doctor-patient trust and mobile medical app adoption. The research findings offer comprehensive insights into doctor-patient trust, the adoption of mobile medical applications, and health information management, providing valuable references for future research and practice in the field.

## 5 Survey instrument development

The UTAUT variables can be traced back to the Unified Theory of Acceptance and Use of Technology (UTAUT) proposed by Venkatesh et al. (1). This theory integrates multiple existing theories of technology acceptance and use, including the Technology Acceptance Model (TAM), Theory of Reasoned Action (TRA), Theory of Planned Behavior (TPB), and other relevant theories. Therefore, the core dimensions of UTAUT, such as Performance Expectancy (PE), Effort Expectancy (EE), Social Influence (SI), and Facilitating Conditions (FC), are derived from these existing research and theoretical foundations.

The survey instrument has been translated into Chinese to facilitate completion by Chinese-speaking users. The translation process was conducted by professional translation services to ensure accuracy and cultural appropriateness. The translated version also underwent validation procedures to assess its linguistic equivalence and to maintain the integrity of the survey instrument.

The sample size is 216, the gender distribution in the sample is 42% male and 58%. The recruitment process for patients participating in the study involved reaching out to individuals diagnosed with the specific condition being studied through healthcare facilities and patient support groups. The selection criteria included individuals who met the defined criteria for the study, and informed consent was obtained from each participant prior to their involvement. The recruitment strategy prioritized reaching out to a diverse range of participants to ensure a representative sample for the research. Ethical considerations were strictly adhered to throughout the recruitment process to protect the rights and privacy of the participants.

### 5.1 Independent variables

The selection of the three situational variables was based on their relevance to the study’s context. These variables, namely “I have experienced a prolonged outbreak lockdown,” “My family has experienced a prolonged outbreak lockdown,” and “My area has experienced a prolonged outbreak lockdown,” were chosen to explore the impact of personal, familial, and community-level experiences during a prolonged outbreak lockdown on the research outcomes. These variables were deemed essential for understanding the situational factors affecting technology adoption and use behavior in the specific context of extended lockdown periods.

PE variable of performance expectation was measured by 3 questions, including “Using mobile medical APP can help improve my health level,” “using mobile medical APP can solve my health and medical needs,” “using mobile medical APP can save my time in obtaining medical services.”

The EE variable of performance expectation was measured by three questions, including “I think mobile medical APP is very useful,” “It will not take me a long time to learn how to use mobile medical APP,” “It is easy to obtain health and medical resources using mobile medical APP.”

Social impact SI variable was measured by 3 questions, including “My family members think I should use mobile medical APP,” “My friends think I should use mobile medical APP,” “My colleagues (classmates) think I should use mobile medical APP.”

The FC variable of the promotion condition was measured by three questions, including “My local network is stable and I can freely use the mobile medical APP,” “My mobile network is stable and I can freely use the mobile medical APP,” and “the mobile medical APP is compatible with my mobile phone and I can use it anytime and anywhere.”

### 5.2 Adjusting the variables

Doctor-patient trust was measured by six questions, including “The doctor will put my needs first,” “I will follow the doctor’s advice,” “What the general doctor tells me is true,” “trust the doctor’s judgment of my condition,” “I think the doctor will take everything into account when diagnosing and treating,” and “If there is a mistake in the diagnosis, the doctor will let me know.”

### 5.3 Dependent variables

The use intention of the dependent variable BI was measured by three questions, including “I am willing to use the mobile medical APP again,” “I am willing to recommend the mobile medical APP to others,” and “I will give a positive evaluation of the mobile medical app.”

### 5.4 Research hypothesis

**Figure 1:**
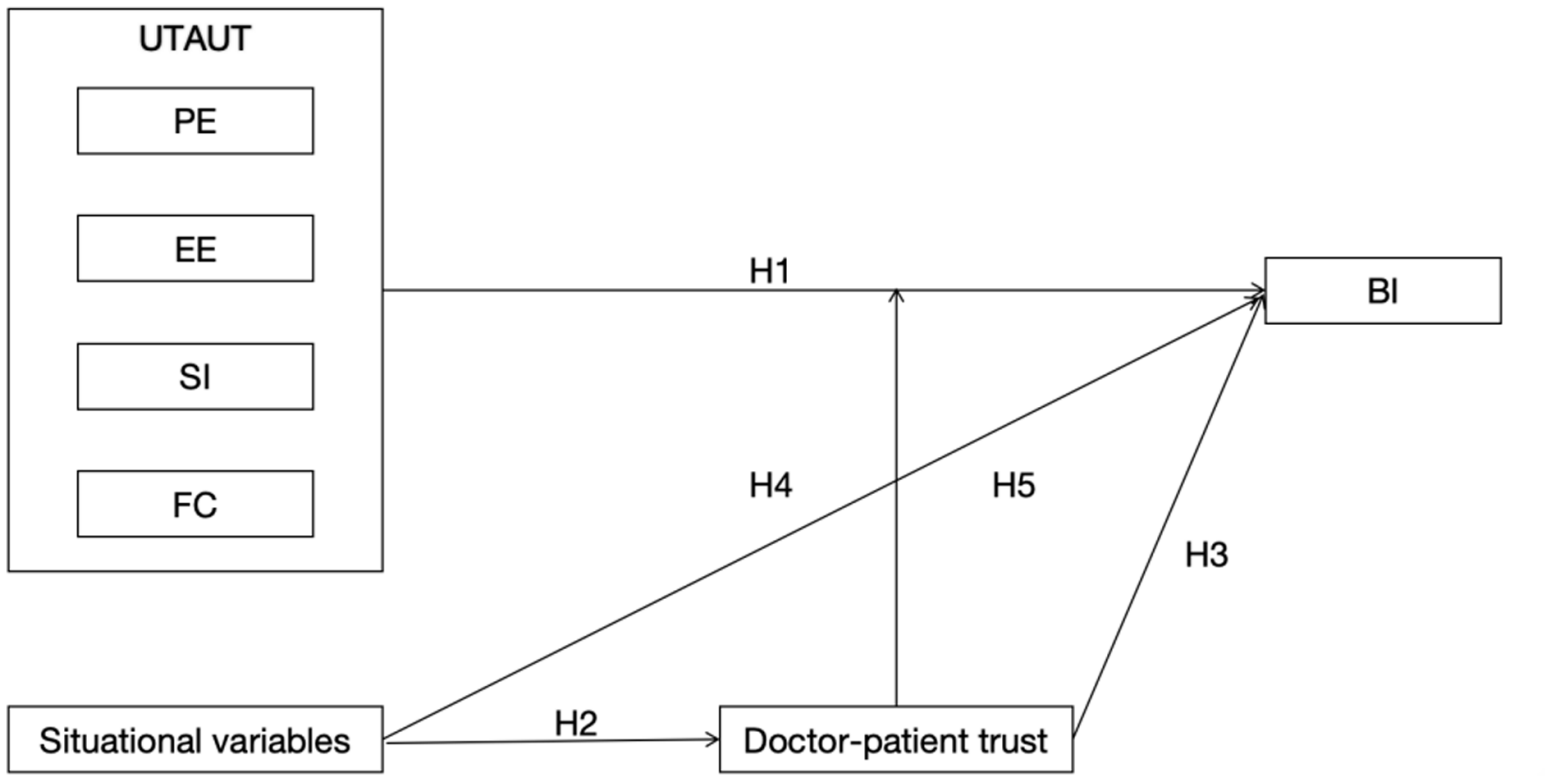
Research model.

The research model, as illustrated in Figure 1, proposes the following hypotheses:

*H1a:* Performance expectations have a positive impact on users’ intention to use mobile medical applications.

*H1b:* Effort expectations have a positive impact on users’ intention to use mobile medical applications.

*H1c:* Social influence has a positive impact on users’ intention to use mobile medical applications.

*H1d:* Facilitating conditions have a positive impact on users’ intention to use mobile medical applications.

*H2:* The epidemic has a positive impact on doctor-patient trust.

*H3:* Doctor-patient trust has a positive impact on users’ intention to use mobile medical applications.

*H4:* Doctor-patient trust moderates the relationship.

*H5:* The epidemic has a positive impact on users’ intention to use mobile medical applications.

## 6 Quantitative analysis

### 6.1 Reliability and validity test

As can be seen from the following reliability and validity data, independent variables, situation variables, PE, EE, social influence, promotion conditions, mediating variables, doctor-patient trust, and use intention of dependent variables all pass the reliability and validity test and can be used as the basis for subsequent analysis (Tables 1, 2).

**Table 1:** Reliability analysis table of continuous variables.

Cronbach reliability analysis of continuous variables		
Serial number	Variables	Cronbach α coefficient
1	Situational variables	0.956
2	PE	0.910
3	EE	0.887
4	SI	0.956
5	FC	0.943
6	Doctor-patient trust	0.933
7	BI	0.942

**Table 2:** Continuous variable validity analysis table.

Continuous variable validity analysis table			
Serial number	variable	Cumulative variance explanation rate after rotation	KMO value
1	Situational variables	91.935%	0.778
2	PE	84.779%	0.752
3	EE	81.732%	0.737
4	SI	91.997%	0.766
5	FC	89.857%	0.707
6	Doctor-patient trust	75.785%	0.879
7	BI	89.672%	0.763

### 6.2 Principal component analysis

The Table 3 illustrates the information extraction status of the principal components for the research items, as well as the corresponding relationships between the principal components and the research items. From the table, it can be observed that the communalities values for all research items are higher than 0.4, indicating a strong association between the research items and the principal components, enabling effective extraction of information.

**Table 3:** Principal component analysis.

Loadings coefficient table		
Name	Loadings coefficients	Communality (common factor variance)
	Principal component 1	
PE	0.904	0.818
FC	0.839	0.704
SI	0.879	0.772
EE	0.952	0.907
BI	0.902	0.814
Doctor-patient trust	0.818	0.670

### 6.3 Regression analysis

In the linear regression analysis conducted in this study, Performance Expectancy (PE), Effort Expectancy (EE), Social Influence (SI), and Facilitating Conditions (FC) were considered as independent variables, while Behavioral Intention (BI) was the dependent variable. The formulated model is represented as BI = 0.206 + 0.149*PE + 0.108*EE + 0.312*SI + 0.381*FC, with an R-square value of 0.786, indicating that PE, EE, SI, and FC collectively explain 78.6% of the variance in BI. The model successfully passed the F test (*F* = 193.786, *p* = 0.000 < 0.05), signifying that at least one of the independent variables significantly impacts BI. Despite the detection of multicollinearity (VI*F* value >5 but <10), suggestions for addressing collinearity include utilizing ridge regression or stepwise regression. It is recommended to further examine variables with close correlations and consider their removal for a refined analysis.

Specifically, the regression coefficient for PE is 0.149 (*t* = 2.536, *p* = 0.012 < 0.05), indicating a significant positive influence on BI. For EE, the regression coefficient of 0.108 (*t* = 1.434, *p* = 0.153 > 0.05) suggests no significant impact on BI. Conversely, the coefficient for SI is 0.312 (*t* = 5.724, *p* = 0.000 < 0.01), implying a significant positive association with BI. Similarly, FC demonstrates a significant positive impact on BI, with a coefficient of 0.381 (*t* = 8.269, *p* = 0.000 < 0.01). In summary, the analysis reveals that PE, SI, and FC positively influence BI, while EE does not exhibit a significant effect.

The concluded model indicates that PE, SI, and FC play pivotal roles in determining BI, emphasizing their significance in shaping users’ behavioral intentions towards mobile health management services (Table 4).

**Table 4:** Results of linear regression analysis.

Results of linear regression analysis (*n* = 216)									
	Non-normalized coefficient		Coefficient of standardization	*t*	*p*-value	VIF	*R* ^2^	Adjust *R*^2^	*F*
	*B*	Standard error	*Beta*						
Constant	0.206	0.126	–	1.639	0.103	–	0.786	0.782	*F* (4,211) = 193.786, *p* = 0.000
PE	0.149	0.059	0.152	2.536	0.012 *	3.534			
EE	0.108	0.075	0.109	1.434	0.153	5.698			
SI	0.312	0.055	0.323	5.724	0.000 * *	3.143			
FC	0.381	0.046	0.405	8.269	0.000 * *	2.370			

Dependent variable: BI; D-W value: 2.190; * *p* < 0.05, ** *p* < 0.01.

The linear regression analysis in this study was conducted with doctor-patient trust as the independent variable and Behavioral Intention (BI) as the dependent variable. The model is represented as: BI = 1.738 + 0.552* Doctor-patient trust, with an R-square value of 0.399, indicating that doctor-patient trust can account for 39.9% of the variation in BI. The model successfully passed the F-test (*F* = 141.899, *p* = 0.000 < 0.05), confirming that doctor-patient trust significantly influences BI. Further analysis revealed that the regression coefficient for doctor-patient trust is 0.552 (*t* = 11.912, *p* = 0.000 < 0.01), indicating a significant positive impact on BI.

In summary, the analysis underscores that doctor-patient trust plays a crucial role in shaping users’ Behavioral Intention (BI), with a substantial positive influence. These findings highlight the importance of fostering trust in the doctor-patient relationship to enhance users’ intentions towards utilizing mobile health management services (Table 5).

**Table 5:** Results of linear regression analysis.

Results of linear regression analysis (*n* = 216)									
	Non-normalized coefficient		Coefficient of standardization	*t*	*p*-value	VIF	*R* ^2^	Adjust *R*^2^	*F*
	*B*	Standard error	*Beta*						
Constant	1.738	0.161	–	10.809	0.000 * *	–	0.399	0.396	*F* (1,214) = 141.899, *p* = 0.000
Doctor-patient trust	0.552	0.046	0.631	11.912	0.000 * *	1.000			

Dependent variable: BI; D-W value: 1.980; * *p* < 0.05, ** *p* < 0.01.

In the conducted linear regression analysis, the independent variable was the situational variable, while the dependent variable was doctor-patient trust. The model formula calculated was: Doctor-patient trust = 2.008 + 0.381 * situational variable. The R-square value of the model indicated to be 0.289, implying that situational variables can account for 28.9% of the variance in doctor-patient trust. The F-test conducted on the model demonstrated statistical significance (*F* = 86.851, *p* = 0.000 < 0.05), confirming that the situational variables significantly influence doctor-patient trust.

Further analysis revealed that the regression coefficient for the situational variables is 0.381 (*t* = 9.319, *p* = 0.000 < 0.01), signifying a significant positive impact on doctor-patient trust.

Overall, the summary analysis concludes that all situational variables have a significant positive effect on doctor-patient trust. These findings emphasize the substantial impact of situational factors on the development of trust in the doctor-patient relationship (Table 6).

**Table 6:** Results of linear regression analysis.

Results of linear regression analysis (*n* = 216)									
	Non-normalized coefficient		Coefficient of standardization	*t*	*p*-value	VIF	*R* ^2^	Adjust *R*^2^	*F*
	*B*	Standard error	*Beta*						
Constant	2.008	0.153	–	13.135	0.000 * *	–	0.289	0.285	*F* (1,214) = 86.851, *p* = 0.000
Situational variables	0.381	0.041	0.537	9.319	0.000 * *	1.000			

Dependent variable: doctor-patient trust; D-W value: 1.838; * *p* < 0.05, ** *p* < 0.01.

In the conducted linear regression analysis, the independent variable was the situational variable, while the dependent variable was Behavioral Intention (BI). The model formula derived from the analysis is: BI = 2.884 + 0.199 * situational variable. The R-square value of the model, at 0.103, indicates that the situational variable can account for 10.3% of the variance in BI. Upon conducting the F-test on the model, statistical significance was observed (*F* = 24.667, *p* = 0.000 < 0.05), affirming that the situational variable indeed impacts BI.

Further analysis revealed that the regression coefficient for the situational variable is 0.199 (*t* = 4.967, *p* = 0.000 < 0.01), indicating a significant positive influence on BI.

Overall, the summary analysis underscores that all situational variables hold a significant positive impact on BI. These findings highlight the importance of situational factors in influencing individuals’ Behavioral Intentions and emphasize the role of context in shaping decision-making processes (Table 7).

**Table 7:** Results of linear regression analysis.

Results of linear regression analysis (*n* = 216)									
	Non-normalized coefficient		Coefficient of standardization	*t*	*p*-value	VIF	*R* ^2^	Adjust *R*^2^	*F*
	*B*	Standard Error	*Beta*						
Constant	2.884	0.150	–	19.229	0.000 * *	–	0.103	0.099	*F* (1,214) = 24.667, *p* = 0.000
Situational variables	0.199	0.040	0.321	4.967	0.000 * *	1.000			

Dependent variable: BI; D-W value: 1.790; * *p* < 0.05, ** *p* < 0.01.

### 6.4 Adjustment effect analysis

The analysis of the adjustment effect is segmented into three models in this study. Model 1 incorporates the independent variable (SI), Model 2 introduces the moderating variable (doctor-patient trust) in addition to Model 1, and Model 3 further includes the interaction term (product term of the independent variable and the moderating variable) on top of Model 2.

In Model 1, the objective is to investigate the impact of the independent variable (SI) on the dependent variable (BI) without considering the influence of the moderating variable (doctor-patient trust). The results displayed in the table reveal that the independent variable (SI) exhibits statistical significance (*t* = 19.744, *p* = 0.000 < 0.05), indicating a substantial influence of SI on BI.

The examination of the moderating effect entails two approaches: evaluating the significance of the change in *F*-value from Model 2 to Model 3, and assessing the significance of the interaction terms in Model 3. In this instance, the moderating effect is analyzed utilizing the second approach.

The table data indicates that the interaction between SI and doctor-patient trust demonstrates statistical significance (*t* = −2.458, *p* = 0.015 < 0.05), signifying that when SI exerts an impact on BI, the moderating variable (doctor-patient trust) significantly influences the amplitude of the impact at different levels (Table 8).

**Table 8:** Results of moderating effect analysis.

Results of moderating effect analysis (*n* = 216)															
	Model 1					Model 2					Model 3				
	*B*	Standard error	*t*	*p*-value	Beta	B	Standard error	*t*	*p*-value	*Beta*	*B*	Standard error	*t*	*p*-value	*Beta*
Constant	3.580	0.034	106.303	0.000 * *	–	3.580	0.033	109.551	0.000 * *	–	3.628	0.038	96.494	0.000 * *	–
SI	0.777	0.039	19.744	0.000 * *	0.803	0.655	0.050	13.138	0.000 * *	0.678	0.667	0.050	13.471	0.000 * *	0.691
Doctor-patient trust						0.170	0.045	3.779	0.000 * *	0.195	0.163	0.045	3.641	0.000 * *	0.186
SI* Doctor-patient trust											0.091	0.037	2.458	0.015 *	0.096
*R* ^2^	0.646					0.668					0.677				
Adjust *R*^2^	0.644					0.665					0.672				
*F* number	*F* (1,214) = 389.815, *p* = 0.000					*F* (2,213) = 214.140, *p* = 0.000					*F* (3,212) = 148.153, *p* = 0.000				
△*R*^2^	0.646					0.022					0.009				
△*F* value	*F* (1,214) = 389.815, *p* = 0.000					*F* (1,213) = 14.278, *p* = 0.000					*F* (1,212) = 6.041, *p* = 0.015				

Dependent variable: BI; * *p* < 0.05, ** *p* < 0.01.

The interaction terms of the other three independent variables PE, EE, FC and doctor-patient trust do not show significance, which means that when PE, EE, FC have an influence on BI, the influence amplitude of the regulating variable (doctor-patient trust) remains the same at different levels.

### 6.5 Ridge regression analysis

Ridge Regression analysis is used to address multicollinearity issues in linear regression analysis by introducing k identity matrix units to estimate regression coefficients. This may result in information loss, yet facilitate reasonable estimation of the regression model.

The process includes three main steps:

1. Prior to the analysis, confirm the value of K through ridge trace plots.
2. Select the smallest K value where the standardized regression coefficients of each independent variable stabilize. A smaller K value indicates less bias, with K = 0 representing ordinary least squares (OLS) linear regression. SPSSAU provides recommendations for K values or users can subjectively identify and select them.
3. Once K value is determined, it can be inputted to obtain the Ridge Regression model estimation.

**Figure 2:**
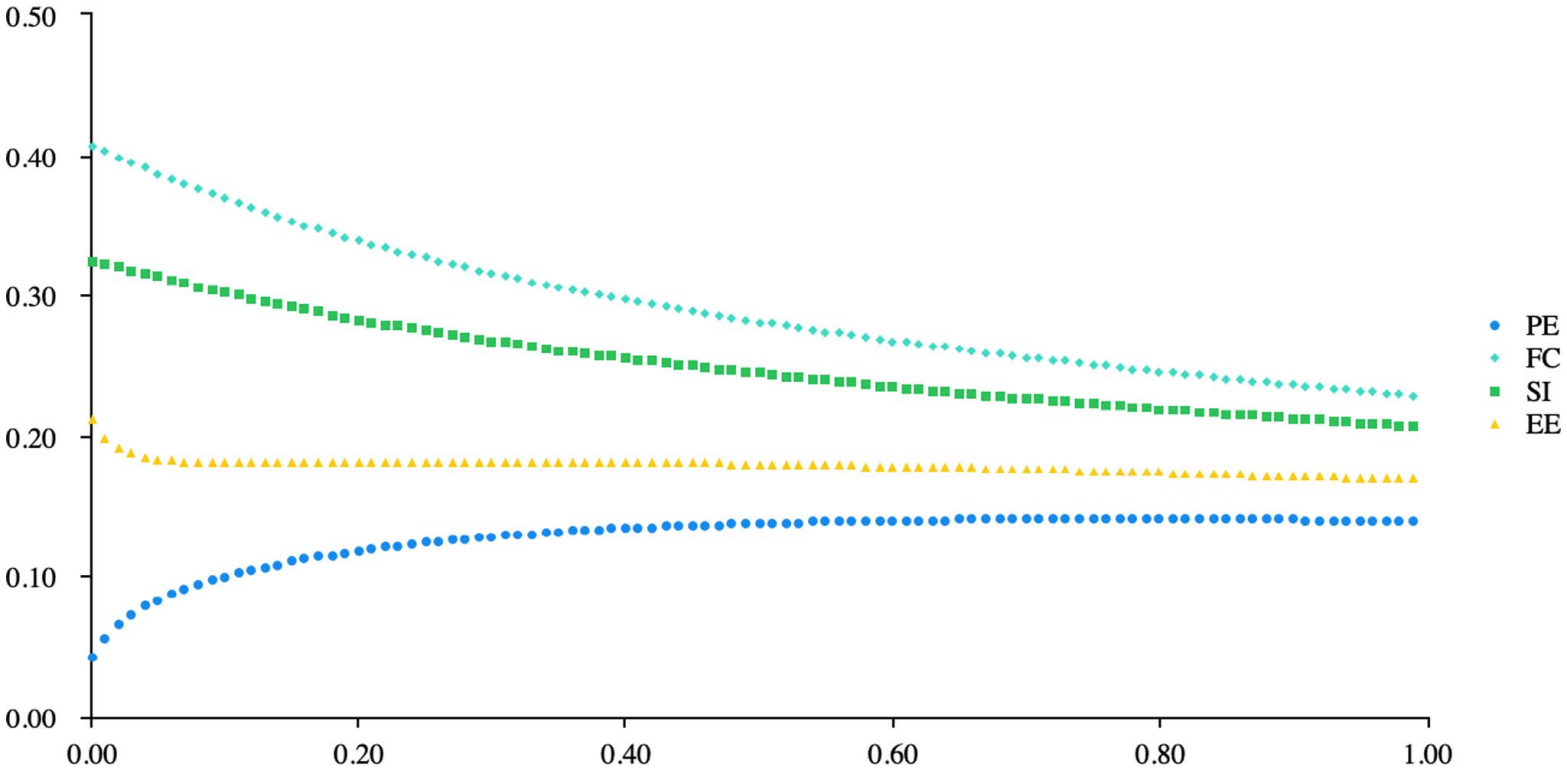
Ridge trace.

In the analysis, when PE, FC, SI, and EE are independent variables and Trust is the dependent variable, the ridge trace plot visually indicates the optimal K value. Users can leverage the recommended K value provided by SPSSAU, based on criteria including VIF < =10 and a preference for smaller K values, suggesting K = 0.02 (Figure 2).

From the Table 9, it can be observed that in the Ridge regression analysis, PE, FC, SI, and EE were used as independent variables, while BI was used as the dependent variable, with a K value of 0.020. The model’s R-squared value of 0.786 indicates that PE, FC, SI, and EE can explain 78.59% of the variance in BI. The F-test results showed that the model passed the F-test (*F* = 193.647, *p* = 0.000 < 0.05), indicating that at least one of PE, FC, SI, or EE has a significant effect on BI. The model formula is represented as: BI = 0.228 + 0.064*PE + 0.374*FC + 0.309*SI + 0.197*EE. The regression coefficient for PE was 0.064 (*t* = 0.863, *p* = 0.389 > 0.05), indicating that PE does not have a significant effect on BI. The regression coefficient for FC was 0.374 (*t* = 8.778, *p* = 0.000 < 0.01), suggesting that FC has a significant positive influence on BI. Similarly, the regression coefficient for SI was 0.309 (*t* = 6.342, *p* = 0.000 < 0.01), signifying a significant positive effect on BI, while the coefficient for EE was 0.197 (*t* = 2.195, *p* = 0.029 < 0.05), indicating a significant positive impact of EE on BI. In conclusion, it can be inferred that FC, SI, and EE have significant positive effects on BI, while PE does not have a significant influence on BI.

**Table 9:** Ridge regression analysis.

Ridge regression analysis results						
	Unstandardized coefficients		Standardized coefficients	*t*	*p*-value	VIF value
	*B*	Standard error	*Beta*			
Constant	0.228	0.125	–	1.828	0.069	–
PE	0.064	0.074	0.065	0.863	0.389	5.665
FC	0.374	0.043	0.398	8.778	0.000**	2.027
SI	0.309	0.049	0.320	6.342	0.000**	2.509
EE	0.197	0.090	0.192	2.195	0.029*	7.510
*R* ^2^	0.786					
Adjusted *R*^2^	0.782					
*F*	*F* (4,211) = 193.647, *p* = 0.000					

Dependent variable: BI; * *p* < 0.05, ** *p* < 0.01.

### 6.6 Lasso regression analysis

From the Table 10, it can be observed that, using PE, FC, EE, and SI as independent variables and BI as the dependent variable for Lasso Regression analysis, the value of K is set at 0.010. The model’s R-squared value is 0.748, indicating that PE, FC, EE, and SI can explain 74.76% of the variance in BI. Upon conducting an F-test for the model, it was found that the model passed the F-test (*F* = 156.229, *p* = 0.000 < 0.05), suggesting that at least one of PE, FC, EE, or SI has an impact on BI. The model equation is: BI = 0.956 + 0.000*PE + 0.299*FC + 0.199*EE + 0.241*SI.

**Table 10:** Lasso regression analysis.

Lasso regression analysis					
	Unstandardized coefficients		Standardized coefficients	*t*	*p*-value
	*B*	Standard error	*Beta*		
Intercept	0.956	0.136	–	7.024	0.000**
PE	0.000	0.099	0.056	0.000	1.000
FC	0.299	0.048	0.402	6.232	0.000**
EE	0.199	0.122	0.198	1.636	0.103
SI	0.241	0.056	0.322	4.347	0.000**
*R* ^2^	0.748				
Adjusted *R*^2^	0.743				
*F*	*F* (4,211) = 156.229, *p* = 0.000				

Dependent variable: BI; * *p* < 0.05, ** *p* < 0.01.

The regression coefficient value for PE is 0.000 (*t* = 0.000, *p* = 1.000 > 0.05), indicating that PE does not have an impact on BI. The regression coefficient value for FC is 0.299 (*t* = 6.232, *p* = 0.000 < 0.01), indicating that FC has a significant positive impact on BI. The regression coefficient value for EE is 0.199 (*t* = 1.636, *p* = 0.103 > 0.05), indicating that EE does not have an impact on BI. The regression coefficient value for SI is 0.241 (*t* = 4.347, *p* = 0.000 < 0.01), indicating that SI has a significant positive impact on BI. In summary, the analysis suggests that FC and SI have a significant positive impact on BI, while PE and EE do not have an impact on BI.

Based on the linear regression analysis, PE, EE, SI, and FC collectively account for 78.6% of the variability in BI, with the model passing the F-test, indicating the presence of at least one significant predictor for BI. Notably, while PE shows a non-significant influence on BI, both SI and FC have significant positive effects. These findings underscore the pivotal roles of SI and FC in determining BI.

In the Ridge regression analysis, PE, FC, SI, and EE explain 78.59% of the variance in BI, with the model passing the F-test. FC, SI, and EE demonstrate significant positive impacts on BI, whereas PE shows no significant effect. This aligns with the linear regression results, emphasizing the influences of FC, SI, and EE on BI.

Conversely, the Lasso regression analysis reveals that PE, FC, EE, and SI account for 74.76% of the variance in BI, with the model also passing the F-test. FC and SI exhibit significant positive effects on BI, while PE and EE do not show significant impacts. Though differing slightly from the linear and Ridge regression outcomes, the results reaffirm the significance of FC and SI in relation to BI.

By comparing the results from the three distinct regression methods, a comprehensive understanding of the impact of each predictor variable on the dependent variable can be gained. This aids in identifying key factors influencing behavioral intentions and offers deeper insights into the research problem. Ultimately, integrating findings from multiple analysis methods can lead to more robust and insightful conclusions, enhancing the depth and foresight of the study.

### 6.7 Conclusion of quantitative analysis

*H1a:* Performance Expectancy was identified to have a significant positive impact on users’ intention to use mobile medical apps, providing robust support for this hypothesis.

*H1b:* In contrast to expectations, Effort Expectancy was found to have a non-significant impact on users’ Behavioral Intention (BI) towards mobile medical apps in the conducted analyses.

*H1c:* Social Influence exhibited a significant positive influence on users’ BI towards mobile medical apps, thereby confirming this hypothesis.

*H1d:* Facilitating Conditions demonstrated a substantial positive effect on users’ intention to utilize mobile medical apps, offering empirical validation for this hypothesis.

*H2:* The Epidemic condition had a positive effect on doctor-patient trust, which yielded significant results.

*H3:* Doctor-patient trust was identified to positively influence users’ intention to use mobile medical apps based on the results.

*H4:* The moderating effect on doctor-patient trust partially supported the hypothesis, indicating some impact on users’ behavioral intentions.

*H5:* Moreover, the Epidemic condition was found to positively influence users’ intention to utilize mobile therapy apps, which was statistically significant in the testing.

These findings provide valuable insights into the dynamics between user perceptions, external factors such as the epidemic, doctor-patient trust, and the intention to use mobile medical and therapy applications.

## 7 Qualitative analysis

The study participants consisted of 30 individuals from various groups, including 10 mobile medical app enterprise managers, 10 dedicated doctors associated with mobile medical apps, and 10 registered users of mobile medical apps. These participants were selected from prominent cities such as Shanghai, Beijing, and Guangzhou to ensure a diverse representation.

### 7.1 Analysis of the impact of the epidemic on doctor-patient trust

During the COVID-19 epidemic, a significant shift in the doctor-patient relationship has been observed. News reports indicate that, unlike the conventional tense and disharmonious relationship, medical staff have treated patients as family members, and patients have viewed medical staff as benefactors. This unprecedented harmony has resulted in an ideal doctor-patient relationship characterized by intimacy, harmony, beauty, and ideal interactions.

In contrast to the prevalent lack of trust between doctors and patients in modern times, patients often harbor suspicions regarding excessive examinations, overmedication, unnecessary surgeries, poor technical skills, and questionable medical ethics. However, the major crisis presented by COVID-19 has fostered a shared sense of destiny between doctors and patients. Confronting a common threat, only by collaborating and fighting together can they overcome the epidemic. In hospitals, patients exhibit a high degree of trust in medical staff and recognize their dedicated service, leading to positive interactions between the two parties.

Additionally, compared to regular healthcare settings, where patient satisfaction tends to be relatively low, makeshift hospitals provide equal access to medical resources regardless of patients’ status or social standing. This egalitarian approach contributes to patients’ elevated sense of medical experience and satisfaction.

During the epidemic, medical workers have gained widespread attention and recognition from society. The media portrays their professional responsibilities and shares touching stories, positioning them as “national heroes.” Within makeshift hospitals, the doctor-patient relationship has reverted to its original simplicity, with patients showing respect towards medical staff, and medical staff providing attentive care. By fulfilling their professional duties, medical workers not only achieve the goal of curing diseases and saving lives but also experience an enhanced sense of professional fulfillment.

### 7.2 Deep causes of doctor-patient trust

#### 7.2.1 The doctor-patient trust crisis as a key driver of tension

In the current doctor-patient relationship, especially in large hospitals such as the well-known top three hospitals, overcrowding and long waiting times have become prevalent. Doctors face immense work pressure, and their medical service attitudes often fall short of expectations. Patients, during their medical journey, often find themselves in a relatively powerless and subordinate position, lacking equality and the right to participate in active dialogues. Their rights and interests are not adequately reflected or respected, leading to distrust in medical technology, ethics, suspicions of doctors’ diagnostic and treatment behaviors, and doubts about their motivations. Consequently, medical staff endure not only the high risks and uncertainties of medical practices but also the psychological confusion accompanying patients’ lack of understanding, cooperation, and respect. This culminates in the crisis of trust between doctors and patients, resulting in tense doctor-patient relationships. The intensified tension further aggravates the trust crisis, perpetuating disharmony within the medical environment and creating a vicious cycle.

In makeshift hospitals during the epidemic, doctors and patients are interconnected within a community of shared destiny. Patients exhibit trust and respect towards medical staff, while medical staff reciprocate with trust and care. This positive interaction fosters a harmonious and friendly state in the doctor-patient relationship, promoting stable trust between the two parties. Ultimately, the doctor-patient relationship revolves around the interactions between individuals. Harmonious coexistence between people depends on mutual trust, as distrust engenders disharmony and potential conflicts. The foundation of a harmonious doctor-patient relationship lies in mutual trust between doctors and patients. Without trust, tension emerges between the two parties. Therefore, the contemporary crisis of doctor-patient trust serves as a significant catalyst for tension between doctors and patients.

#### 7.2.2 The potential of mobile medical apps in mitigating the deterioration of doctor-patient trust in the context of asymmetric medical resources

In the qualitative research, it became evident that the asymmetry of medical resources significantly contributed to the deterioration of doctor-patient trust. Patients’ experiences of limited access to medical professionals, long waiting times, and a perceived lack of attention and respect led to heightened frustration and skepticism. However, the emergence of mobile medical apps presents a promising solution to alleviate this issue and potentially restore trust in the doctor-patient relationship.

Mobile medical apps offer a convenient and accessible platform for patients to seek medical services and information, irrespective of their geographical location or the availability of healthcare resources. By utilizing these apps, patients can connect with healthcare providers, schedule appointments, receive consultations, and access reliable health information at their fingertips. Such seamless access to medical services helps address the asymmetry in medical resources, as patients are no longer solely reliant on physical proximity to healthcare facilities.

Moreover, mobile medical apps empower patients to take an active role in their healthcare. Patients can track and manage their health conditions, access personalized health recommendations, and easily communicate with healthcare professionals. This active participation not only enhances patients’ sense of control and involvement but also fosters a more collaborative doctor-patient relationship.

By bridging the gap between patients and healthcare providers, mobile medical apps have the potential to restore and enhance trust. Patients can perceive these apps as a reliable source of medical information, care, and support, thereby reducing their skepticism and concerns. The convenience and efficiency offered by mobile medical apps also contribute to a positive healthcare experience, improving patients’ satisfaction and overall perception of the quality of care received.

However, it is important to acknowledge the need for measures that ensure the privacy and security of patient data in mobile medical apps. Confidentiality and trust in handling sensitive health information are critical to establish and maintain doctor-patient trust. Healthcare organizations and app developers must implement robust security protocols and adhere to ethical guidelines to safeguard patient information.

In conclusion, the advent of mobile medical apps presents a promising means to address the asymmetry of medical resources and mitigate the deterioration of doctor-patient trust. These apps offer convenient access to medical services, empower patients to take an active role in their healthcare, and facilitate collaboration between patients and healthcare providers. By leveraging the power of technology to bridge gaps and improve healthcare accessibility, mobile medical apps hold potential for restoring trust and fostering a more balanced and patient-centric doctor-patient relationship.

### 7.3 Suggestions on improving the trust between doctors and patients

To address the challenges and limitations in the current healthcare landscape, it is imperative to promote the development of “Internet Plus” medical services. This entails leveraging high-tech information technology to enhance various aspects of medical services, including “Internet Plus smart medical,” “Internet Plus telemedicine,” and “Internet Plus new services.” By fully harnessing the potential of advanced information technology, patients can overcome the constraints of time and space, enabling convenient access to medical services.

The integration of smart medical technologies and the internet allows for remote consultations, diagnosis, and treatment, enabling patients to receive quality healthcare services regardless of their geographical location. Telemedicine, facilitated by digital platforms and mobile medical apps, fills the gap in medical resources by providing virtual medical consultations and delivering healthcare remotely. This helps to address the issue of limited access to healthcare professionals, especially in remote or underserved areas.

Furthermore, the development of “Internet Plus new services” expands the scope of medical services beyond traditional models. It encompasses innovative approaches such as online health management, wearable health monitoring devices, and predictive analytics. These advancements enable individuals to actively engage in their own healthcare, empowering them to monitor their health conditions, receive timely alerts, and make informed choices.

The promotion of “Internet Plus” medical services and the widespread adoption of mobile medical apps present significant opportunities for improving healthcare accessibility, efficiency, and quality. However, it is crucial to ensure that these advancements are implemented in a manner that prioritizes patient privacy and data security. Robust regulatory frameworks and ethical guidelines must be put in place to safeguard patient information and maintain public trust in these digital healthcare services.

The vigorous development of “Internet Plus” medical services, encompassing smart medical technologies, telemedicine, and innovative models, holds immense potential for transforming healthcare delivery. By embracing digital advancements and mobile medical apps, healthcare systems can bridge the gap in medical resources, enhance patient access to quality care, and empower individuals to take an active role in their own health management. However, careful attention must be given to privacy and security concerns to ensure the responsible and ethical implementation of these technologies.

## 8 Conclusion and discussion

The findings of this study highlight the significant impact of Performance Expectancy, Social Influence, and Facilitating Conditions on users’ intention to use mobile medical apps, while emphasizing the crucial role of doctor-patient trust, particularly during an epidemic. These results provide valuable insights for optimizing the promotion and utilization of mobile healthcare applications, underscoring the importance of doctor-patient trust in fostering user acceptance and utilization. Future research should delve deeper into the interactions of these factors and develop effective strategies to further enhance the usage of mobile healthcare applications.

A notable observation is the tangible impact of pandemic circumstances in positively influencing the dynamics of trust between doctors and patients. This revelation showcases the transformative potential of crisis situations in reshaping doctor-patient relationships and cultivating heightened levels of trust. Healthcare organizations are urged to leverage insights garnered during the pandemic to formulate enduring strategies that strengthen and uphold trust between healthcare providers and patients in the post-pandemic era.

Furthermore, the study illuminates the substantial positive impact of trust between healthcare stakeholders and patients during the epidemic, offering valuable lessons for healthcare institutions and public health agencies. The exceptional performance and dedicated professionalism exhibited by healthcare workers during the outbreak have laid a robust foundation for fortifying doctor-patient relationships. Proactive measures taken by healthcare institutions during the crisis, such as providing complimentary healthcare services and promoting shared risks between doctors and patients, have further solidified the bond of trust between healthcare providers and patients.

In response to these positive transformations, healthcare organizations are urged to develop enduring strategies and policies to nurture and reinforce trust between healthcare workers and patients post-epidemic. Operational strategies could include enhancing communication channels, fostering trust-centered healthcare paradigms, and implementing mechanisms for continuous improvement and feedback facilitation to ensure personalized attention and support for patients throughout their healthcare journey.

By translating these encouraging shifts into tangible interventions for ongoing enhancement, healthcare institutions can elevate trust between healthcare stakeholders and patients, ultimately enhancing the quality and efficacy of healthcare services. Such initiatives cultivate an inclusive and collaborative healthcare environment, laying a strong foundation for bolstering patient satisfaction, health outcomes, and overall healthcare services.

The study’s findings underscore the pivotal role of trust between doctors and patients in moderating social influence within the realm of mobile healthcare applications. By positioning trust as a critical moderator, the results advocate for strategies focused on nurturing trust to amplify the efficacy of social influence in driving the adoption of mobile healthcare services. Integrating trust-centric components in promotional strategies, such as transparent communication, personalized interactions, and dependable service provisions, holds the promise of enhancing the impact of social factors on users’ behavioral intentions. These insights highlight the paramount importance of navigating trust dynamics in crafting targeted and compelling promotional campaigns for mobile healthcare applications, ultimately fostering increased user engagement and robust adoption rates.

In conclusion, this study sheds light on the factors influencing users’ intent to continue using mobile medical apps, underscoring the defining role of trust between doctors and patients in this domain. These insights deepen our understanding of the mobile health app landscape and provide actionable guidelines for app developers, healthcare entities, and policymakers to improve user acceptance and facilitate effective utilization of these apps.

Acknowledging the limitations of the study, there is a call for further exploration. Potential sources of bias, such as sample representativeness and measurement inaccuracies, could impact the reliability and generalizability of research findings. Future research efforts should focus on broadening sample size and diversity, refining data collection precision, and implementing bias control measures to enhance the scientific rigor and fidelity of the study.

Moving forward, researchers must address the study’s limitations, particularly those related to sample representation and measurement accuracy, to strengthen the reliability and credibility of future research endeavors. Mitigating these limitations through effective measures will propel research progress in this critical domain.

## Data Availability

The original contributions presented in the study are included in the article/Supplementary material, further inquiries can be directed to the corresponding author.
